# Supplementary material for: Liver X Receptor Ligand GAC0001E5 Downregulates Antioxidant Capacity and ERBB2/HER2 Expression in HER2-Positive Breast Cancer Cells
Source: Cancers (Basel). 2024 Apr 25;16(9):1651. doi: 10.3390/cancers16091651 (PMC11083021; doi:10.3390/cancers16091651)
Supplement: Supplementary file 1 [file cancers-16-01651-s001.zip › cancers-2959271-supplementary.pdf]

36B4-F: GTGTTCGACAATGGCAGCAT
36B4-R: GACACCCTCCAGGAAGCGA
SREBP1c-F: GGAGGGGTAGGGCCAACGGCCT
SREBP1c-R: CATGTCTTCGAAAGTGCAATCC
LXRβ-F: ATCAAGAGGCCGCAGGACCA
LXRβ-R: AGGCGAAGACCTGCTCCGAG
ABCA1-F: TGTGAGGCGGGAAAGACAGAG
ABCA1-R: AGCCCAAAGCACTCCACCAGGA
ABCG1-F: CGATGAGCCCACCAGCGGC
ABCG1-R: ACCCCCTTGAGCGAGCCCTT
ACACA (ACC) -F: GCAGGTCACACGTCTCTTTAT
ACACA (ACC) -R: CCAGCCTGTCATCCTCAATATC
FASN-F: ACAGGGACAACCTGGAGTTCT
FASN-R: CTGTGGTCCCACTTGATGAGT
SCD1-F: TTCAGAAACACATGCTGATCCTCATAA
SCD1-R: ATTAAGCACCACAGCATATCGCAAGAA
GLS1-F: TTCCAGAAGGCACAGACATG
GLS1-R: GGCTCAGTACTCTTTCACCAG
GOT1-F: CAACTGGGATTGACCCAACT
GOT1-R: GGAACAGAAACCGGTGCTT
GOT2-F: GTTTGCCTCTGCCAATCATATG
GOT2-R: GAGGGTTGGAATACATGGGAC
GLUD1-F: AGGAATGACACCAGGGTTTG
GLUD1-R: TCAGACTCACCAACAGCAATAC
SLC7A11-F: TTTCTGCATCCACATTCCAA
SLC7A11-R: AACACCATCTGGCATTGTGA
HER1 (ERBB1)-F: AGGCACGAGTAACAAGCTCAC
HER1 (ERBB1)-R: ATGAGGACATAACCAGCCACC
HER2 (ERBB2)-F: TGCAGGGAAACCTGGAACTC
HER2 (ERBB2)-R: ACAGGGGTGGTATTGTTCAGC
HER3 (ERBB3)-F: GGTGATGGGGAACCTTGAGAT
HER3 (ERBB3)-R: CTGTCACTTCTCGAATCCACTG
HER4 (ERBB4)-F: GCCTCTGGAGAATTTACGCAT
HER4 (ERBB4)-R: GGGTTCCGAACAATATCTTGCC

**Supplementary Figure S1.** The list of primers used
